# Assessment of asthma control and asthma exacerbations in the epidemiology and natural history of asthma: outcomes and treatment regimens (TENOR) observational cohort

**DOI:** 10.1007/s13665-012-0025-x

**Published:** 2012-09-20

**Authors:** Bradley E. Chipps, Robert S. Zeiger, Alejandro Dorenbaum, Larry Borish, Sally E. Wenzel, Dave P. Miller, Mary Lou Hayden, Eugene R. Bleecker, F. Estelle R. Simons, Stanley J. Szefler, Scott T. Weiss, Tmirah Haselkorn, TENOR Study Group

**Affiliations:** 1 Capital Allergy and Respiratory Disease Center, Sacramento, 5609 J Street, Suite C, Sacramento, CA 95819 USA; 2 Department of Allergy, Kaiser Permanente SD, 7060 Clairemont Mesa Blvd, San Diego, CA 92111 USA; 3 Genentech, Inc, MS-58B, 1 DNA Way, South San Francisco, CA 94080 USA; 4 Asthma and Allergic Disease Center, University of Virginia, Charlottesville, VA 22908 USA; 5 Pulmonary, Allergy and Critical Care Medicine, University of Pittsburgh Asthma Institute, NW 931 Montefiore, 3459 Fifth Ave, Pittsburgh, PA 15213 USA; 6 ICON Clinical Research, 188 The Embarcadero # 200, San Francisco, CA 94105 USA; 7 University of Virginia Employee Health, P.O. Box 800398, Charlottesville, VA 22908 USA; 8 Center for Genomics and Personalized Medicine Research, Wake Forest University School of Medicine, Medical Center Blvd, Winston-Salem, NC 27157 USA; 9 Department of Pediatrics and Child Health, Department of Immunology, University of Manitoba, 820 Sherbrook St, Winnipeg, Manitoba, Canada R3A 1R9; 10 Department of Pediatrics, National Jewish Health and University of Colorado School of Medicine, 1400 Jackson Street, Denver, CO 80206 USA; 11 Channing Laboratory, Brigham and Women’s Hospital, Harvard Medical School, 181 Longwood Avenue, Boston, MA 02115 USA

**Keywords:** Severe asthma, Difficult-to-treat asthma, Asthma control, Exacerbation

## Abstract

Patients with severe or difficult-to-treat asthma account for substantial asthma morbidity, mortality, and healthcare burden despite comprising only a small proportion of the total asthma population. TENOR, a multicenter, observational, prospective cohort study was initiated in 2001. It enrolled 4,756 adults, adolescents and children with severe or difficult-to-treat asthma who were followed semi-annually and annually for three years, enabling insight to be gained into this understudied population. A broad range of demographic, clinical, and patient self-reported assessments were completed during the follow-up period. Here, we present key findings from the TENOR registry in relation to asthma control and exacerbations, including the identification of specific subgroups found to be at particularly high-risk. Identification of the factors and subgroups associated with poor asthma control and increased risk of exacerbations can help physicians design individual asthma management, and improve asthma-related health outcomes for these patients.

## Introduction

According to World Health Organization (WHO) estimates, 235 million people worldwide currently have asthma [1]. Although patients with severe or difficult-to-treat asthma comprise only a small proportion (<15 %) of the total asthma population, they account for substantial asthma morbidity, mortality, and healthcare costs [2–6]. As for all patients with asthma, the primary objective of treatment for patients with severe disease is to achieve and maintain clinical control [7••]. To improve outcomes among these patients, it is necessary to understand the extent to which this objective is being met, identify barriers that may hinder achievement of asthma control, and ascertain whether specific subgroups of patients are at increased risk of poor control.

Large registries that accumulate data from “real-world” settings are a valuable tool with which to evaluate treatment outcome. In 2001, a multicenter, observational, prospective cohort study, The Epidemiology and Natural History of Asthma: Outcomes and Treatment Regimens (TENOR), was initiated to study patients described by their physicians as having severe or difficult-to-treat asthma [8, 9]. Data from TENOR have been reported in more than 25 publications, and provide insight into asthma-related health outcome and barriers to optimum asthma care for these patients. Analyses of TENOR data have also provided the opportunity to characterize patients with severe or difficult-to-treat asthma with regard to atopy. For example, in TENOR most patients were found to have high immunoglobulin (IgE) levels, particularly males, children, smokers, non-white racial or ethnic group, and adults with childhood-onset disease [10]. In young patients, IgE levels were found to be associated with asthma severity [10], and high frequencies of comorbid allergic diseases and allergen sensitization were seen [11].

In this review, we summarize the findings from TENOR in relation to asthma control and asthma exacerbations in patients with severe or difficult-to-treat disease and further examine specific patient subgroups within this population.

## Defining asthma severity and control

When TENOR was initiated in 2001, asthma severity was defined in the National Heart Lung and Blood Institute (NHLBI) guidelines according to symptoms, activity limitation, rescue medication use, and exacerbation frequency [12]. In 2007, these guidelines were updated, recommending that physicians assess asthma severity using two domains: current impairment (day-to-day symptoms and limitation of activity) and future risk (likely outcome if patient remains on current treatment, based on asthma severity, history, and probability of exacerbation) [13, 14]. Similarly, current Global Initiative for Asthma (GINA) guidelines note that asthma severity encompasses both the underlying nature of the disease and the response to treatment [7••]. Consequently, the GINA guidelines classify asthma as severe if it requires high intensity treatment or if control is not achieved despite such treatment. Both NHLBI and GINA guidelines also recognize the importance of evaluating future risk of exacerbation, on the basis of current clinical control, lung function, and exacerbation history [7••, 13].

Data from the TENOR registry have proved extremely useful for identifying patients at high-risk of poor asthma control and frequent asthma exacerbations. This review summarizes the analyses and findings related to asthma control and exacerbations in TENOR through the evolution of these terms and definitions.

## The TENOR registry

### Objectives and design

The primary objective of TENOR was to collect prospective data to improve our understanding of the natural history of asthma in patients with severe or difficult-to-treat disease [8]. Secondary objectives were to examine the relationship between features of asthma treatment and outcome, to observe the frequency of pre-defined comorbid conditions, and to describe the relationship between IgE levels and disease.

**Fig. 1:**
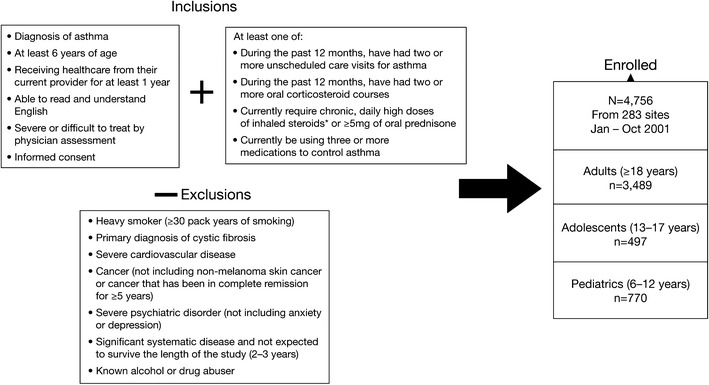
TENOR study design [15•]. Adapted from Chipps et al. 2012 [15•]; copyright (2012), with permission from Elsevier. *Asterisk*, daily high doses of inhaled steroids were defined by the American Thoracic Society refractory asthma guidelines for adults [37] and by the 1997 National Heart Lung and Blood Institute (NHLBI) guidelines for children [12]

The TENOR study design has been described in detail elsewhere [8] and is shown in Fig. 1. TENOR is a large, observational study of severe or difficult-to-treat asthma which enrolled 4,756 patients (3,489 adults ≥18 years, 497 adolescents 13–17 years, and 770 children 6–12 years) with diverse racial and ethnic backgrounds, and from different geographic areas [8]. Patients were enrolled from specialized asthma care settings in the USA, including managed care organizations, community physicians, group practices, and academic centers.

Patients were included in the TENOR registry if they had severe or difficult-to-treat asthma, with asthma severity being determined by their physician. Patients with mild or moderate asthma were therefore eligible for enrollment if their asthma was considered difficult-to-treat. Patients were assessed as having “difficult-to-treat” asthma on the basis of pre-specified criteria, including the need for multiple drugs, occurrence of frequent exacerbations, severe exacerbations, inability to avoid asthma triggers, and use of a complex treatment regimen [8]. Patients were also required to have high use of the healthcare system (≥2 unscheduled care visits for asthma or ≥2 oral corticosteroid (OCS) courses) or high medication use (requiring ≥3 medications to control asthma or requiring long-term daily high-doses of inhaled corticosteroids (ICS)) in the previous 12 months. At enrollment, 48 % of patients had severe asthma, 48 % moderate asthma, 3 % mild asthma, and 96 % difficult-to-treat asthma. After enrollment, patients completed seven study visits over three years, during which a range of demographic, clinical, standard laboratory, and patient self-reported assessments was completed. Baseline demographics and clinical characteristics of the TENOR population are presented in Table 1.

**Table 1:** TENOR baseline demographics and clinical characteristics [8]

Variable	Overall	Adults (≥18 years)	Adolescents (13–17 years)	Children (6–12 years)
Patients, *n* (%)	4756 (100)	3489 (73.36)	497 (10.45)	770 (16.19)
Age, mean ± SD, years	38.9 ± 20.92	48.9 ± 14.85	14.5 ± 1.34	9.5 ± 1.88
Weight, mean ± SD, kg	75.2 ± 26.53	83.9 ± 2.22	66.9 ± 21.03	41.1 ± 16.64
BMI, mean ± SD, kg/m^2^	28.3 ± 8.59	30.4 ± 7.73	25.4 ± 9.86	20.7 ± 6.17
IgE, geometric mean, IU/mL	106.6	85.2	223.8	182.5
Sex, *n* (%)				
Female	2945 (62.2)	2475 (71.2)	213 (42.9)	257 (33.5)
Male	1792 (37.8)	999 (28.8)	283 (57.1)	510 (66.5)
Race or ethnicity, *n* (%)				
White	3555 (75.1)	2769 (79.8)	323 (65.4)	463 (60.4)
Black	712 (15.0)	404 (11.6)	115 (23.2)	193 (25.2)
Hispanic	303 (6.4)	197 (5.7)	36 (7.3)	70 (9.1)
Asian or Pacific Islander	72 (1.5)	57 (1.6)	7 (1.4)	8 (1.0)
Other	91 (1.9)	44 (1.2)	14 (2.8)	33 (4.3)
Physician assessment of severity, *n* (%)				
Mild	149 (3.2)	91 (2.6)	19 (3.8)	39 (5.1)
Moderate	2285 (48.4)	1585 (46.1)	237 (47.9)	453 (59.1)
Severe	2285 (48.4)	1771 (51.2)	239 (48.3)	275 (35.9)
Smoking history, *n* (%)				
Never smoked	3454 (73.1)	2207 (63.7)	483 (97.8)	764 (99.6)
Past smoker	1113 (23.6)	1110 (32.0)	3 (0.6)	0
Current smoker	159 (3.4)	148 (4.3)	8 (1.6)	3 (0.4)
Predicted pre-bronchodilator FEV_1_, *n* (%)				
≤60 %	1015 (23.5)	893 (27.8)	69 (15.7)	53 (8.0)
>60 % to <80 %	1292 (29.9)	1015 (31.6)	104 (23.7)	173 (26.0)
≥80 %	2007 (46.5)	1302 (40.6)	266 (60.6)	439 (66.0)
Pre-bronchodilator FEV_1_, (% predicted), mean ± SD	77.2 ± 23.28	74.2 ± 23.45	84.0 ± 21.63	87.0 ± 19.57
Post-bronchodilator FEV_1_ (% predicted), mean ± SD	82.5 ± 23.04	79.0 ± 23.08	91.1 ± 21.03	93.9 ± 18.50
QoL score^a^, mean	NA	4.6	5.2	5.4

Adapted from Dolan et al. 2004 [8]; copyright (2004), with permission from Elsevier

^a^Asthma-related QoL measured using the Juniper Mini Asthma Quality of Life Questionnaire for patients ≥13 years and the Pediatric Asthma Quality of Life Questionnaire with Standardized Activities for patients aged 6–12 years

BMI, body mass index; FEV_1_, forced expiratory volume in 1 second; IgE, immunoglobulin E; NA, not applicable; QoL, quality of life; SD, standard deviation; TENOR, the epidemiology and natural history of asthma: outcomes and treatment regimens

At baseline, at least one asthma hospitalization or emergency department (ED) visit in the previous three months occurred in 5 % and 15 % of adults, 10 % and 17 % of adolescents, and 9 % and 22 % of children, respectively. Almost half of adults and children, and ~40 % of adolescents reported OCS courses and unscheduled visits for asthma in the previous three months [8]. Across all age groups, approximately 10 % of patients reported a history of intubation for asthma. This high healthcare utilization was observed despite the use of multiple long-term controller medications [8, 9]. Notably, of those using at least three long-term controllers, 15 % of adults, 19 % of adolescents, and approximately 25 % of children had visited an ED in the three months before baseline [15•].

### Recent and future asthma exacerbation

**Fig. 2:**
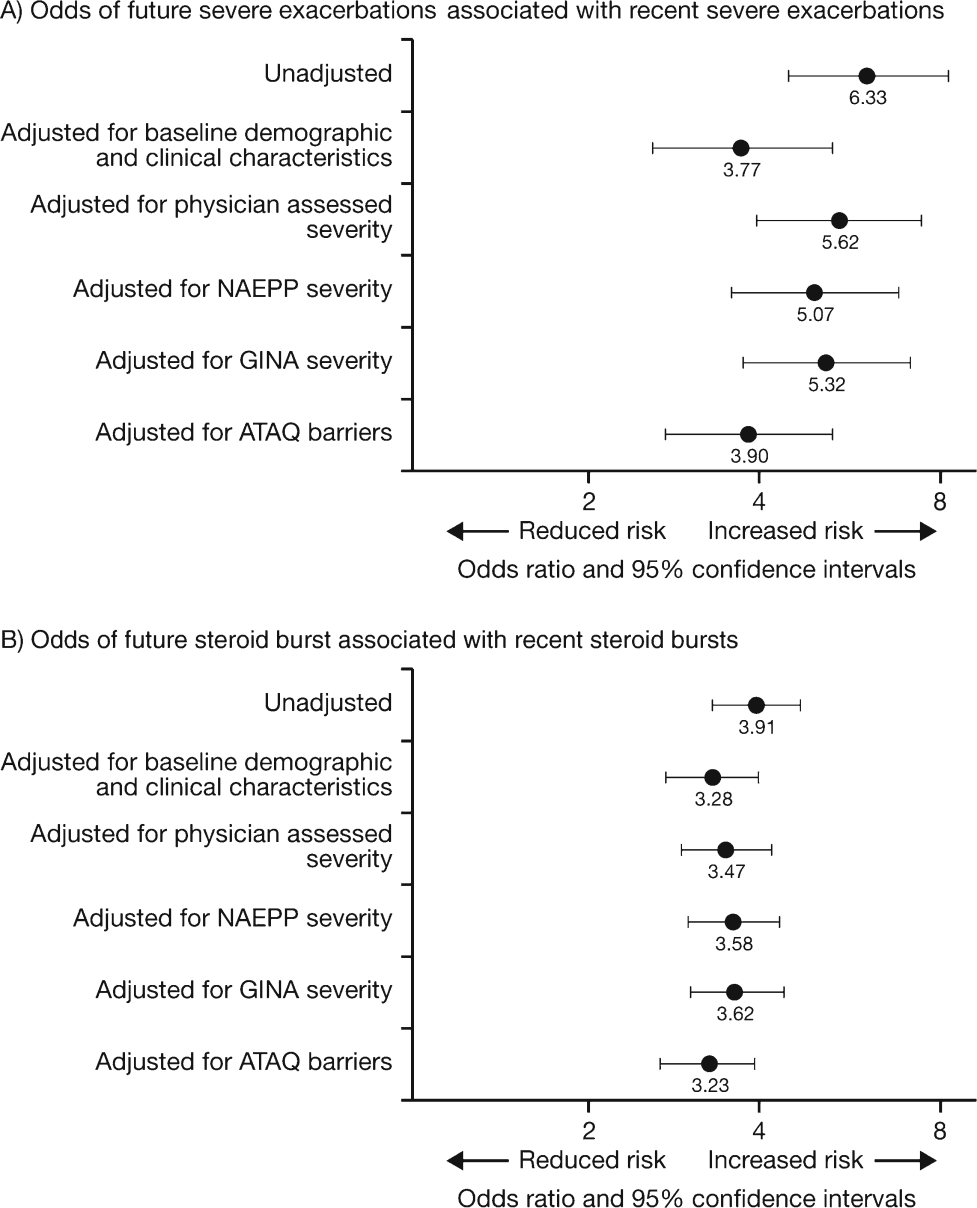
A) Odds of future exacerbations associated with recent exacerbations, B) Odds of future steroid burst associated with recent steroid bursts, adjusted for demographics, asthma severity, and asthma control [16]. Adapted from Miller et al. 2007 [16]; copyright (2007), with permission from Elsevier. Recent referred to the three months before baseline. Severe exacerbations was defined as either an asthma-related emergency department visit or hospitalization. X axis is on a logarithmic scale. *ATAQ*, asthma therapy assessment questionnaire; *GINA*, global initiative for asthma; *NAEPP*, national asthma education and prevention program

Given the high rates of healthcare utilization in the TENOR cohort, subsequent analysis has focused on identifying specific predictors for future asthma exacerbations. In adults, adolescents, and children, after assessing a broad range of variables, recent asthma exacerbation was the strongest predictor of future asthma exacerbation [16]. In patients aged ≥12 years, those who reported an asthma-related ED visit or overnight hospitalization in the three months before baseline were more than sixfold more likely (odds ratio (OR) = 6.33; 95 % confidence interval (CI) 4.57, 8.76), to experience a future severe asthma exacerbation (defined as an asthma-related ED visit, overnight hospitalization, or an asthma-related death) at the 18-month follow-up (Fig. 2) [16]. After adjusting for demographic and clinical factors, the risk of future severe exacerbation remained high (OR = 3.77; 95 % CI 2.62, 5.43). When analysis was individually adjusted for various asthma severity measures, the increased risk was consistently more than fivefold (physician-assessed: OR = 5.62; 95 % CI 4.03, 7.83; National Asthma Education and Prevention Program guideline assessed: OR = 5.07; 95 % CI 3.62, 7.11; GINA guideline assessed: OR = 5.32; 95 % CI 3.80, 7.47). When adjusted for asthma control (using the asthma therapy assessment questionnaire; ATAQ), the risk of future severe exacerbation was nearly fourfold (OR = 3.90; 95 % CI 2.77, 5.50). An increased risk of future exacerbation requiring an OCS course was also seen if patients had a recent exacerbation requiring an OCS course in the three months before baseline (Fig. 2) [16].

Similarly, separate analysis for children (aged 6–11 years) assessed the occurrence and association of a recent severe exacerbation defined as one or more OCS courses during the three months before each of three annual visits (baseline, Month 12, and Month 24), and a future severe exacerbation, defined as one or more OCS courses six or twelve months later. A future severe exacerbation at six months was most strongly predicted by a recent severe exacerbation (OR = 3.08; 95 % CI 2.21, 4.28) and having 3 or 4 allergic triggers (OR = 2.05; 95 % CI 1.31, 3.20) [17•] (Table 2, Model II). Other predictors of a future severe exacerbation at six months included non-white race (OR = 1.77; 95 % CI 1.25, 2.51) and very poorly controlled asthma, as defined by the NHLBI guidelines (OR = 1.59; 95 % CI 1.14, 2.23).

**Table 2:** Predictors of future exacerbations in children aged 6–11 years [17•]

Predictor^a^	Model I^b^			Model II^c^		
	Stepwise model where future exacerbation includes 6-month and 12-month time frames			Additional stepwise reduction of model including only 6-month events		
	OR	95 % CI	*p* value	OR	95 % CI	*p* value
Recent exacerbation	1.99	1.51, 2.61	<0.001	3.08	2.21, 4.28	<0.001
Non-white vs. white	1.76	1.34, 2.32	<0.001	1.77	1.25, 2.51	0.001
Allergic triggers^d^						
1 or 2 allergic triggers	1.39	0.99, 1.95	0.059	1.26	0.82,1.93	0.29
3 or 4 allergic triggers	2.01	1.40, 2.89	<0.001	2.05	1.31, 3.20	0.002
NHLBI guidelines control impairment domain^e^						
VPC vs. NWC	1.40	1.08, 1.80	0.010	1.59	1.14, 2.23	0.007
WC vs. NWC	0.89	0.45, 1.75	0.73	0.85	0.37, 1.92	0.69
Duration of asthma (per year)	1.06	1.01, 1.12	0.021	–	–	–
Non-allergic triggers^f^, 1–2 vs. 0	1.52	1.07, 2.16	0.019	–	–	–

Adapted from Haselkorn et al. 2009c [17•]; copyright (2009), with permission from Elsevier

^a^The stepwise model candidate predictors were age, duration of asthma, sex, white/non-white, obesity, number of long-term controllers, allergic triggers, non-allergic triggers, passive smoking, NHLBI 2007 asthma control (impairment domain), and median income in zip code. Variables that are components of the NHLBI guidelines impairment domain (e.g. ATAQ control problems, spirometry and symptoms) were included only as components of the NHLBI definition

^b^The event of interest (future severe exacerbation) was observed in 469 assessments of 255 patients

^c^ The event of interest (future severe exacerbation) was observed in 250 assessments of 186 patients

^d^Patients with no triggers are the reference group. Allergic triggers include pollen, moldy/musty places, animals, and dust and are based on the question, “Symptoms of asthma are a result of …”

^e^As NWC and WC are not meaningfully different, a single OR was computed for VPC vs. all others, and was used to calculate the predicted probabilities (OR 1.62; 95 % CI 1.16, 2.25, *p* = 0.004)

^f^Non-allergic triggers include emotional stress and cold/sinus infection.

ATAQ, Asthma Therapy Assessment Questionnaire; CI, confidence interval; NHLBI, National Heart, Lung and Blood Institute; NWC, not well controlled; OR, odds ratio; VPC, very poorly controlled; WC, well controlled

This analysis for adults, adolescents, and children reveal that a recent severe exacerbation (within the previous three months) is an important independent predictor of a future severe exacerbations in severe or difficult-to-treat asthma.

### Asthma control and asthma exacerbations

**Fig. 3:**
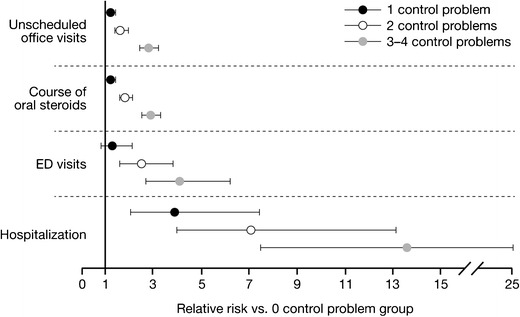
Adjusted risk of acute asthma-related healthcare events by baseline level of asthma control [18]. Adapted from Sullivan et al. 2007 [18]. Asthma control was determined at baseline by use of The Asthma Therapy Assessment Questionnaire. *ED*, emergency department

We also examined the relationship between asthma control and asthma exacerbations. The findings consistently revealed that poor asthma control, whether defined by the patient (using self-reported, validated instruments) or by national asthma guideline classifications, was strongly associated with an increased risk of asthma exacerbations. In an early analysis, TENOR used the ATAQ in patients ≥18 years to assess the association between baseline asthma control, and subsequent severe asthma-related healthcare events within the first year of follow-up. After adjustment for covariates, patients with poor asthma control at baseline (three or four control problems) were found to be at increased risk of: unscheduled office visits (relative risk (RR) = 2.8; 95 % CI 2.4, 3.2), requiring a course of OCS (RR = 2.9; 95 % CI 2.5, 3.3), ED visits (RR = 4.1; 95 % CI 2.7, 6.2), and hospitalization (RR = 13.6; 95 % CI 7.4, 24.9) within the first year of follow-up, compared with patients with no asthma control problems [18] (Fig. 3).

**Fig. 4:**
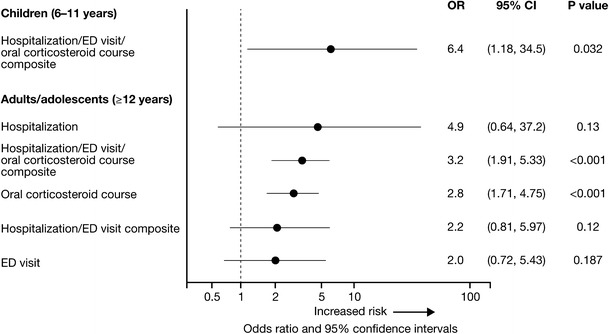
Risk of asthma exacerbations at the Month 30 visit associated with consistently very poorly controlled asthma, as defined by impairment domain of the NHLBI guidelines [19•]. Adapted from Haselkorn et al. 2009 [19•]; copyright (2009), with permission from Elsevier. Final adjusted models for hospitalization and ED visits include previous hospitalization or ED visits, number of long-term controllers, body mass index, allergic triggers, non-allergic triggers, percent predicted FVC, race or ethnicity, and age. Final adjusted models for corticosteroid courses include previous corticosteroid course, chronic obstructive pulmonary disease, non-allergic triggers, percent predicted FEV_1_/FVC ratio, race or ethnicity, and age. *CI*, confidence interval; *ED*, emergency department; *FEV*_*1*_, forced expiratory volume in 1 second; *FVC*, forced vital capacity; *NHLBI*, National Heart Lung and Blood Institute

After the 2007 update to the NHLBI asthma guidelines [13], we evaluated the classification scheme for asthma control based on the impairment domain of the guidelines for identifying patients at risk of future asthma exacerbation [19•]. Using data representing all components of the impairment domain at baseline, Month 12, and Month 24, patients were categorized into two groups: those whose asthma was consistently very poorly controlled from baseline through two years of follow-up, and those whose asthma improved from very poorly controlled at baseline with improvement maintained throughout two years of follow-up. In multivariate analysis, pediatric patients whose asthma was consistently very poorly controlled were at sixfold increased risk of hospitalization, ED visit, or OCS burst (OR = 6.4; 95 % CI 1.2, 34.5) at the Month 30 visit, compared with those who improved over the two-year period. Adult and adolescent patients who were consistently very poorly controlled were more likely to require an OCS course (OR = 2.8; 95 % CI 1.7, 4.8), or have a hospitalization, ED visit, or OCS course when these outcomes were assessed as a composite measure (OR = 3.2; 95 % CI 1.9, 5.3; Fig. 4). These data from TENOR provided validation for the impairment domain of the 2007 NHLBI guidelines as an effective tool for classifying and managing patients with severe or difficult-to-treat asthma.

The NHLBI asthma guidelines were further investigated by assessing the affect of individual components of the impairment domain on the classification of asthma control and prediction of asthma exacerbations. It was found that omission of individual components of the impairment domain resulted in asthma control being wrongly classified in 11–39 % of patients [20•]. The strongest predictor of an asthma exacerbation at 12 months was a previous exacerbation at baseline (in adults and adolescents: OR = 2.93; 95 % CI 2.38, 3.61 and in children: OR = 2.94; 95 % CI 1.71, 5.07). In addition, very poorly controlled asthma, classified on the basis of use of short-acting β_2_-agonists (SABAs), was associated with a twofold higher exacerbation risk in children (OR = 2.03; 95 % CI 1.17, 3.52). In adults and adolescents, significant predictors of exacerbations at Month 12 were very poorly controlled asthma on the basis of lung function (OR = 1.66; 95 % CI 1.34, 2.06) and very poorly controlled asthma on the basis of the ATAQ control score (OR = 1.94; 95 % CI 1.56, 2.40). In addition, for adults and adolescents, very poorly controlled asthma and not well controlled asthma, classified on the basis of the use of SABAs, was also associated with increased exacerbation risk (OR = 1.49; 95 % CI 1.19, 1.85). This study reaffirmed that a recent asthma exacerbation is the strongest predictor of a future exacerbation and that, although specific components of the NHLBI impairment domain may be more important than others in predicting asthma exacerbations, the combined use of the components is essential for improving the identification of patients at high-risk of future exacerbations [20•].

### Asthma control and asthma exacerbations in high-risk patient subgroups

The TENOR registry has provided a valuable opportunity to evaluate asthma control and exacerbations in several patient subgroups, including older patients (≥65 years) who are often excluded from clinical trials. Characterization of these subgroups can assist physicians in addressing individual disease-management needs, and may help to improve asthma-related health outcome.

Compared with younger patients (18–64 years) in TENOR, lower healthcare resource utilization, better asthma-related quality of life (QoL), and fewer asthma control problems were observed for older patients, despite their poorer lung function [21]. These findings may be related to more intensive treatment (as evidenced by greater use of ICS and OCS) and higher medication adherence among older TENOR patients. Also, although older patients reported fewer problems controlling their asthma (*p* < 0.001), they reported worse communication with their physicians (*p* = 0.02) than younger patients.

Body mass index (BMI) and weight have both been positively correlated with asthma incidence and severity, and negatively associated with response to ICS [22–24]. For adult TENOR patients (aged ≥18 years), body weight changes were found to affect asthma control and exacerbation [25]. Patients who gained at least 5 lb (2.27 kg) during a 12-month interval reported poorer asthma control (adjusted OR = 1.22; 95 % CI 1.01, 1.49), worse QoL, and a greater number of OCS courses (OR = 1.31; 95 % CI 1.04, 1.66), than patients who maintained or lost weight.

Studies have shown that African–Americans with asthma are more likely to experience treatment failure than white patients, despite having fewer asthma symptoms and using less rescue medication [26]. It has also been shown that African–Americans tend to have more severe acute asthma episodes than white patients, and have greater utilization of emergency healthcare [26–28]. The underlying reasons for increased asthma morbidity among black patients compared with those from other racial/ethnic groups, have not been fully elucidated. To improve understanding, a TENOR analysis of adult patients (≥18 years) compared 243 black patients with 1,885 white patients. One year after enrollment, black patients were more likely to have a higher frequency of ED visits, more problems with asthma control, poorer QoL, and greater requirement for three or more long-term controllers (among those with physician-assessed severe asthma) [29]. These racial differences were not explained by adjustment for broad sets of confounding variables, including socioeconomics, disease severity, BMI, sensitization to allergens, medication adherence, or treatment setting. Asthma-related behavioral and knowledge factors, which are understudied aspects of current racial disparity research, did not differ between black and white patients. These findings suggest the need to evaluate genetic and pharmacogenetic factors to help explain these racial differences.

Although treatment reverses airway obstruction in patients with asthma, it has been noted that some patients have persistent airflow limitation (PAFL) which is characterized by a lack of reversal of obstruction, despite treatment, and is associated with a high risk of morbidity and mortality [13, 30–32]. A 2007 analysis from the TENOR registry showed that PAFL was highly prevalent in adult (≥18 years) patients with severe or difficult-to-treat asthma, and can be associated with identifiable clinical and demographic characteristics [33]. Of the 1,017 TENOR patients included in this analysis, evidence of PAFL (defined as post-bronchodilator forced expiratory volume in 1 second/forced vital capacity (FEV_1_/FVC) ratio ≤70 % at two annual consecutive visits) was observed for 612 (60 %). Patients with chronic obstructive pulmonary disease (COPD), obesity with a restrictive respiratory pattern, or a ≥30 pack-year history of smoking were excluded. Risk factors for PAFL were old age (OR per 10 years = 1.4; 95 % CI 1.3, 1.6), male gender (OR = 4.5; 95 % CI 2.3, 8.5), black ethnicity (OR = 2.2; 95 % CI 1.3, 2.8), current or past smoking (OR = 3.9; 95 % CI 1.8, 8.6 and 1.6; 95 % CI, 1.2, 2.3, respectively), aspirin sensitivity (OR = 1.5; 95 % CI 1.0, 2.4), and longer asthma duration (OR per 10 years = 1.6; 95 % CI 1.4, 1.8). Factors that appeared to protect against PAFL were Hispanic ethnicity, being better educated, having a history of atopic dermatitis, having pet(s) in the home, and dust sensitivity. Importantly, however, no evidence of PAFL was found for 40 % of patients analyzed, suggesting PAFL may be a distinct asthma phenotype which does not develop in all severe or difficult-to-treat patients with asthma.

It has been noted that patients with aspirin-exacerbated respiratory disease (AERD), which is characterized by aspirin sensitivity, severe persistent asthma, extensive chronic hyperplastic eosinophilic sinusitis, and nasal polyp formation, are at particularly high risk of asthma exacerbations [34, 35]. Aspirin sensitivity as a risk factor for the development of irreversible airway obstruction was evaluated for adult patients (aged ≥18 years) in the TENOR registry [36]. Compared with patients with non-aspirin-sensitive asthma, patients with AERD had significantly lower mean post-bronchodilator percent predicted FEV_1_ (75.3 % vs. 79.9 %), were more likely to have physician-assessed severe asthma (66 % vs. 49 %), to have been intubated for asthma (20 % vs. 11 %), to require OCS courses (56 % vs. 46 %), or require higher doses of ICS (34 % vs. 25 %). These findings suggest that aspirin sensitivity is associated with increased asthma severity, and possible remodeling of the upper and lower airways [36].

## Conclusions

Since its initiation in 2001, the TENOR registry has provided clinicians with unique and important information that can be used to help guide effective management of patients with severe asthma by improved understanding of factors associated with poor asthma control and high levels of healthcare resource use. Analysis of data from the TENOR cohort have revealed that patients with severe or difficult-to-treat asthma should be assessed to determine their recent exacerbation history (previous 3 months). In addition, analyses of data from the TENOR cohort provide strong support for assessment of current asthma control to estimate future exacerbation risk and guide management decisions [19•, 20•]. The most recent NHLBI and GINA guidelines for the management of asthma [7••, 13] both recognize the importance of evaluating future risk of exacerbations on the basis of assessment of exacerbations history and current asthma control.

The TENOR registry has facilitated the identification of several subgroups of patients with severe or difficult-to-treat asthma who are at particular high-risk of poor asthma control and impairment. These include adult (≥18 years) patients who gain weight (≥5 lbs over a 12-month period), black patients, patients with PAFL, and patients with aspirin-sensitive asthma. Increased awareness of these subgroups may help clinicians apply more targeted asthma management strategies to improve asthma-related health outcome for these patients. A summary of the important findings from TENOR discussed in this review, and their clinical implications, are listed in Table 3. However, it should be noted that the findings from TENOR are representative of specialist care in the USA, and therefore might not be generalizable to asthmatic patients in the general population or under primary care management.

**Table 3:** Summary table of important findings from TENOR and applications to patient care

Important findings from the TENOR cohort: Asthma control and exacerbations in patients with severe or difficult-to-treat asthma	Ref.
Patients with severe or difficult-to-treat asthma have poor asthma control and high rates of asthma-related healthcare utilization, despite use of multiple long-term controller medications	[8, 15•]
Poor asthma control, whether defined by self-reported validated instruments, for example the ATAQ, or by use of NHLBI 2007 guideline classifications, is strongly associated with an increased risk of future asthma exacerbations	[18, 19•]
Patients are at significant risk of a future asthma exacerbations if they have had a recent exacerbations in the preceding 3 months	[16, 17•]
Asthma control and exacerbations in high-risk patient subgroups with severe or difficult-to-treat asthma	
Adult (≥18 years) patients who gain weight (≥5 lb during a 12-month period) are more likely to have poorer asthma control, worse QoL, and a greater number of OCS courses compared with patients who maintain or lose weight	[25]
Adult (≥18 years) black patients are at an increased risk of asthma exacerbations, poorer asthma control, and compromised responsiveness to some medications	[29]
PAFL is highly prevalent among adult (≥18 years) patients, particularly those of older age, male sex, black race, who are current or past smokers, aspirin sensitive, or have a long asthma duration	[33]
Adult (≥18 years) patients with aspirin sensitivity are more likely to have severe asthma and possible remodeling of the upper and lower airways	[36]
Applications to patient care	
Recent exacerbations history (preceding 3 months) and an evaluation of the level of asthma control should be included as a component of asthma assessment and management	[16, 17•, 20•]
Future exacerbations for individual patients can be predicted by use of asthma control, defined by the impairment domain of the NHLBI 2007 asthma guidelines	[19•]
Validated, self-assessed measures of asthma control, for example the ATAQ, are effective tools for identifying and managing patients at greatest risk of future health impairment and severe asthma-related incidents	[18]
Physicians should be aware of the importance of good communication with patients, particularly old patients, and ensure appropriate use of ICS and patient adherence to prescribed regimens	[21]
Strategies to prevent weight gain may help patients achieve better asthma control and improve asthma-related QoL	[25]
Increased awareness of patient subgroups that are particularly at risk of poor asthma control and future exacerbations can help in the design of asthma-management strategies for individual patients. These include patients with weight gain, black patients, patients with PAFL, and patients with aspirin-sensitive asthma	[25, 29, 33, 36]

Adapted from Chipps et al. 2012 [15•]; copyright (2012), with permission from Elsevier

ATAQ, Asthma Therapy Assessment Questionnaire; NHLBI, National Heart, Lung and Blood Institute; OCS, oral corticosteroids; PAFL, persistent airflow limitation; QoL, quality of life; TENOR, The Epidemiology and Natural History of Asthma: Outcomes and Treatment Regimens
